# Dosimetric impact of variable air cavity within PTV for rectum cancer

**DOI:** 10.1002/acm2.14539

**Published:** 2024-10-03

**Authors:** Eujin Chan, Simon K. Goodall, Robert Finnegan, Paul Moorfoot, Michael Jameson, Leon Dunn

**Affiliations:** ^1^ GenesisCare Victoria Melbourne Victoria Australia; ^2^ GenesisCare Western Australia Wembley Western Australia Australia; ^3^ School of Physics, Mathematics, and Computing, Faculty of Engineering and Mathematical Sciences University of Western Australia Crawley Western Australia Australia; ^4^ Royal North Shore Hospital Northern Sydney Cancer Centre St Leonards New South Wales Australia; ^5^ GenesisCare New South Wales Alexandria New South Wales Australia; ^6^ University of New South Wales Sydney New South Wales Australia; ^7^ University of Wollongong Wollongong New South Wales Australia

**Keywords:** air cavity expansion, CBCT guidelines, PTV coverage, rectum

## Abstract

**Purpose:**

The aim of this study is to determine the impact of rectal air volume changes on treatment plan quality, and subsequently inform daily cone‐beam computed tomography (CBCT) evaluation constraints, in terms of acceptable rectal air volume during treatment.

**Methods:**

Twelve rectal cancer patients who exhibited rectal air within the PTV on their planning CT were selected. A study was conducted to evaluate the deterioration in plan quality due to expanding air volume. For each case, the air cavity volume was isotropically expanded in three dimensions using predefined margins of 3, 5, 7, and 10 mm, while deforming bladder and rectum contours. A constraint was applied to the bony anatomy to restrict the deformation. Treatment plans were then generated for all twelve patients by recalculating the reference plan with the expanded air cavity volume.

**Results:**

As the air cavity expanded, the maximum relative change in D98% coverage, compared to the reference plan, decreased by 10.8% ± 3.5%, while the D2% increased by 3.5% ± 0.9%. The positioning of the air cavity notably influenced the D98% variability with the 3 mm expansion. D98% coverage falls below 95% when the air cavity volume exceeds 17 cm^3^. On average, D2% coverage increased by 0.5% with each expansion. At the largest expansion, extensive coverage of 102% and 105% isodoses was observed compared to the reference plan.

**Conclusion:**

Air cavity volumes above 17 cm^3^ can potentially degrade the high‐dose PTV coverage while increasing the regions covered by the 102% and 105% isodoses. Clinical CBCT guidelines were deduced, recommending a maximum threshold of 3.2 cm in diameter in any direction.

## 1 INTRODUCTION

The current standard of treatment for patients with locally advanced rectal cancer (LARC) is preoperative chemoradiotherapy (CRT).^1^, ^2^, ^3^ At our institution, the radiation doses prescribed for LARC are 50.4 Gy in 28 fractions or 50 Gy in 25 fractions before radical resection. It is well known that the rectum changes position, volume, and shape between, and, even during fractions of radiotherapy.^4^, ^5^, ^6^, ^7^, ^8^, ^9^, ^10^, ^11^, ^12^, ^13^, ^14^, ^15^ Depending on the volume of air present during treatment, its impact on the rectum size, shape, and surrounding anatomy, can result in complex dose‐volume deposition differences relative to the treatment plan.

Typically, in modern radiotherapy, LARC patients would be treated with intensity‐modulated radiotherapy (IMRT) or volumetric‐modulated arc therapy (VMAT). VMAT and IMRT techniques have the advantage of helping to create highly conformal dose distributions but are more susceptible to daily variation in patient setup and organ motion/deformation.^4–8,15,16^ Image‐guided radiotherapy was employed to assist with these variations. Two‐dimensional (2D) portal imaging is the earliest form of image‐guided radiotherapy which allows patient positioning verification using bone landmarks. In modern radiotherapy, cone‐beam computed tomography (CBCT) is used to analyze the inter‐fraction anatomy differences in 3 dimensions (3D) by comparing the anatomy at the time of treatment to the treatment planning CT.

Soft tissue verification becomes important when internal organs such as the rectum or bladder are subject to size and shape variation. Several studies have been published that focus on changes to the bladder and rectum during fractionated prostate radiotherapy.^4^, ^5^, ^6^, ^8^, ^15^, ^16^ All studies reported that rectum and bladder volume changes affect the position of the prostate which can subsequently lead to cumulative dose variation. However, the evaluation of rectal motion and distention is limited to post‐treatment CBCT analysis.^9^, ^10^, ^11^ The impact of planning dosimetry for varying air cavity rectum patients was limited to the magnetic resonance (MR)‐guided radiotherapy modality.^13^

There have been several studies relating to bladder and rectal inter‐ and intrafraction changes in density that have been published. However, these are mainly related to prostate radiotherapy, as opposed to rectal radiotherapy. Huang et al.^17^ used CBCT images to analyze the changes in both the bladder and rectum during fractionated prostate radiotherapy. The mean percentage differences (± standard deviation) in the volume and radiation dose were 44% (±41) and 18% (±17) for the bladder and 36% (±29) and 22% (±15) for the rectum, respectively. Roeske et al.^18^ also stated that bladder and rectal volumes varied by ±30% according to a treatment planning CT study.

With these volume changes in mind, Daly et al.^12^ evaluated rectal motion via CBCT to determine whether current margin approaches were adequate. Their study reported that, based on a limited number of CBCT scans, the rectum tended to remain within 1.5 cm of the initial location on the treatment planning CT. However, a 1.5 cm margin beyond the posterior bladder edge provides better coverage of the mesorectum than 1 cm for the initial CTV.

In attempting to discern the dosimetric impact of these changes mentioned above, Duman et al.^14^ retrospectively recalculated 22 patients’ VMAT treatment plans on 176 CBCT datasets. Their work found that mean rectal volumes were significantly larger than those estimated at CT and in turn, this resulted in a 9% increase in mean rectum doses. Conversely, mean bladder volumes decreased resulting in a mean increase in dose to the bladder of 8%. Their study concluded that daily changes in bladder and rectal volumes can result in larger actual doses to these organs relative to the planned dose.

This work aimed to assess the impact of rectal air volume changes on treatment plan quality, and subsequently inform daily CBCT evaluation constraints in terms of acceptable rectal air volume changes. The variations in key dosimetric parameters were evaluated for increasing PTV air volumes to guide their clinical acceptability.

## 2 MATERIALS AND METHODS

### 2.1 Patient selection

Twelve patients who were diagnosed with T2 or T3, N0‐N2, and M0 malignant neoplasm of rectum and previously treated with VMAT were selected. All twelve patients were selected from previous clinical cases where the presence of rectal air within the PTV was observed on the planning CT. Eight patients received a total dose of 50.4 Gy to the high‐dose PTV and 45 Gy to the low‐dose PTV for at‐risk lymph nodes, delivered in 28 fractions. The remaining four patients received 50 Gy to the high‐dose PTV and 45 Gy to the low‐dose PTV for at‐risk lymph nodes, delivered in 25 fractions.

### 2.2 Contour deformation

To model the impact of changes in rectal air cavity volume, a framework for simulating anatomical changes was adapted from the open‐source software PlatiPy,^19^ which has previously been used in the context of simulating anatomical changes in head and neck cancer patients.^20^

**FIGURE 1:**
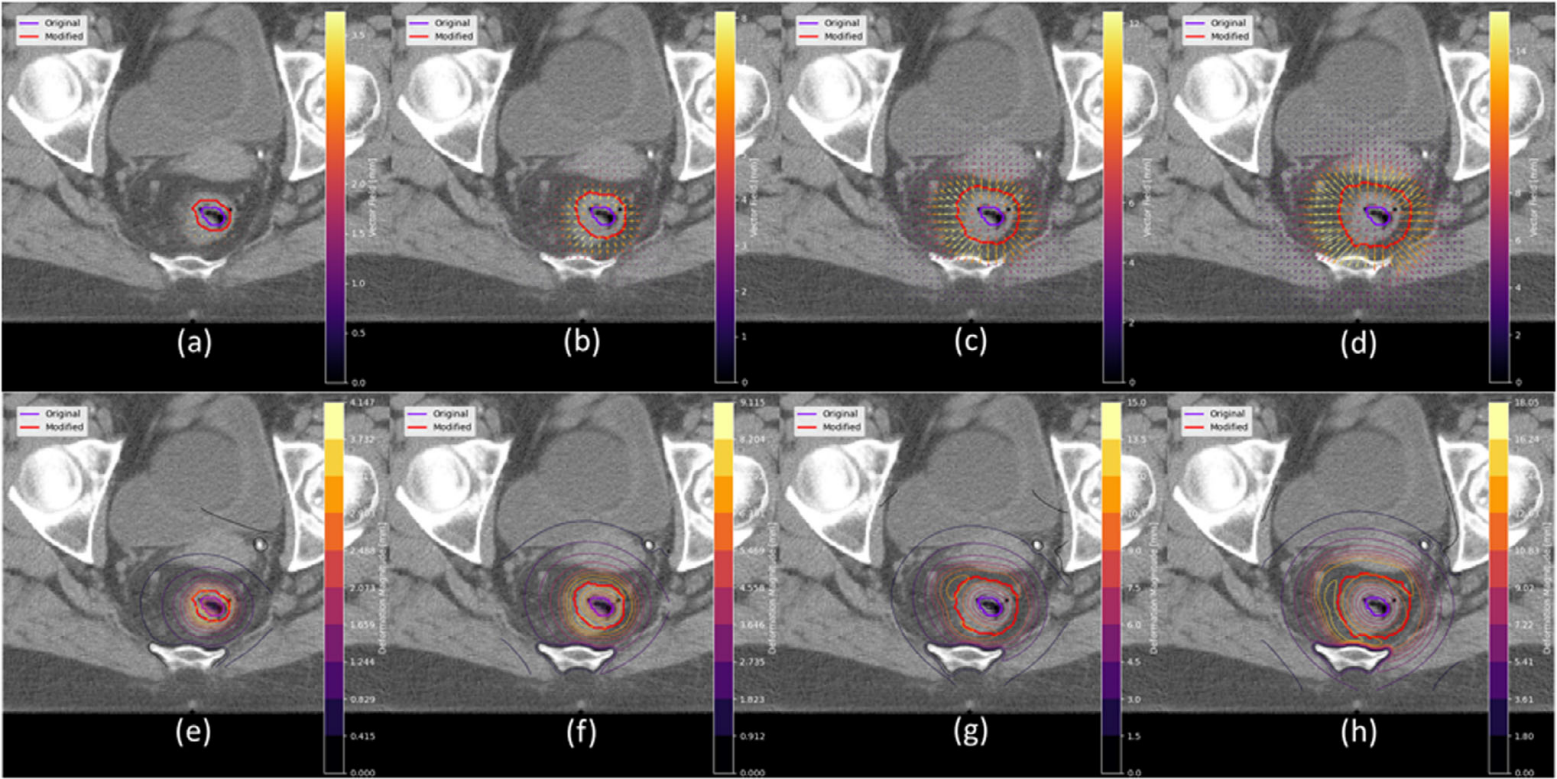
The deformation vector field representing the spatial transformation between the original and modified air cavity contours for expansions of (a) 3 mm, (b) 5 mm, (c) 7 mm, and (d) 10 mm. For the same expansions, (e), (f), (g), and (h) show the magnitude of the deformation vector field, depicted as iso‐contours. The restriction of deformation near the bony anatomy, particularly near the coccyx, is evident. Note that this deformation occurs in three dimensions.

For each patient, the air cavity volume contour was expanded isotropically in all 3D with pre‐defined margins of 3, 5, 7, and 10 mm. A previously described distance‐preserving registration process^21^, ^22^ was then used to calculate a deformation vector field (DVF) to smoothly deform the original air cavity contour to the expanded contour, as illustrated in Figure 1. A constraint to restrict deformation near bony anatomy was applied. No compensation was applied for this restriction, hence the same physical expansion may lead to slight variations in volume changes among different patients, depending on the proximity of the rectal gas volume to the nearby bony anatomy. The effect of this constraint on the deformation is visualized in the bottom row of Figure 1. For each simulated expansion, the resulting DVF was used to deform the bladder and rectum contours, in addition to the rectal gas volume contour.

### 2.3 Treatment planning

In this study, all clinical plans utilized dose control volumes as part of the optimization objectives to regulate hot or cold spots. For comparative purposes, a reference plan was created for all twelve patients using the optimization objectives implemented clinically but excluding the dose control volumes. The reference plans remained clinically acceptable as the dose constraints were within tolerance. All plans were generated within the Eclipse treatment planning system (Varian Medical Systems, Palo Alto, CA) using a 6 MV beam comprising two 360° coplanar arcs and collimator angles set at 15° and 80°. Acuros XB (v. 16.01. Eclipse, Varian Medical Systems, Palo Alto, CA) dose calculation algorithm with a 2.5 mm dose grid was used for calculations. For every air cavity volume, the reference plan was recalculated using the identical Multileaf Collimator (MLC) segments and Monitor Units (MUs), while overriding the air cavity contour to air density (−1000 HU). Dose‐to‐medium dose reporting mode was selected for the Acuros XB dose calculation.

### 2.4 Plan analysis

The plan without air cavity expansion served as the reference plan for comparison. Departmental constraints, outlined in Table 1, were used to evaluate plan quality. For each patient, dosimetric parameters including D98% and D2% (representing the minimum dose received by 98% or 2% of the volume) were collected for the high‐dose PTV from each plan. The dose and volume of the rectum and bladder were recorded to assess the consequences of the air cavity expansions.

**TABLE 1:** Department constraints for rectum patients. PTV4500eval* structure excludes the PTV5040/PTV5000 from PTV4500 for evaluation purposes.

Structure	Criteria
PTV5040 & PTV5000	D98% ≥ 95% D2% ≤ 107%
PTV4500	D98% ≥ 42.75 Gy
PTV4500eval*	D2% ≤ 48.15 Gy
Bowel Cavity	V45Gy ≤ 195 cm^3^ Dmax ≤ 50 Gy
Bladder	V35Gy < 50% V40Gy < 35% V50Gy < 5%
FemHead_Neck_L	V44Gy < 5% V40Gy < 35%
FemHead_Neck_R	V44Gy < 5% V40Gy < 35%
Genitalia_Ex	V20Gy < 50% V30Gy < 35% V40Gy < 5%
IliacCrest	V30Gy < 50% V40Gy < 35% V50Gy < 5%

## 3 RESULTS

### 3.1 Volume evaluation

**FIGURE 2:**
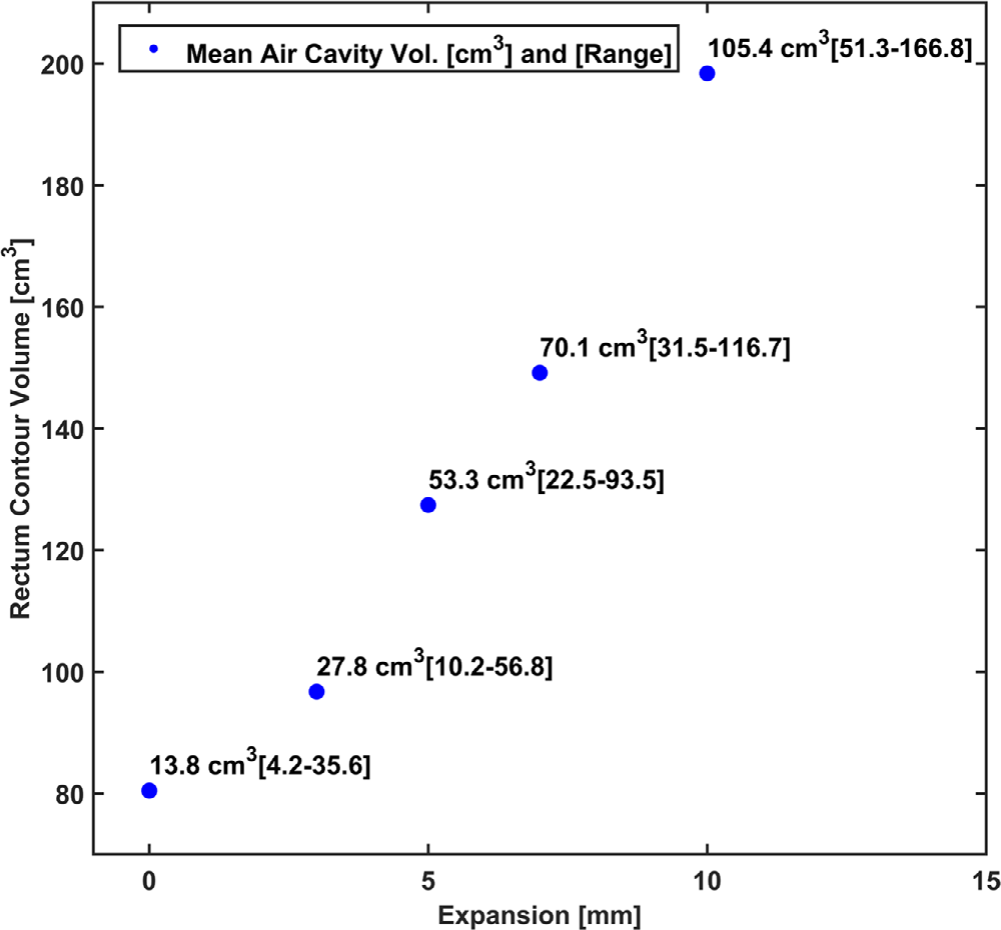
The mean volume of air cavity and rectum contour due to the uniform expansions observed across all subjects in this study. The range of the expanded air cavity volumes is shown in brackets next to the mean value.

**FIGURE 3:**
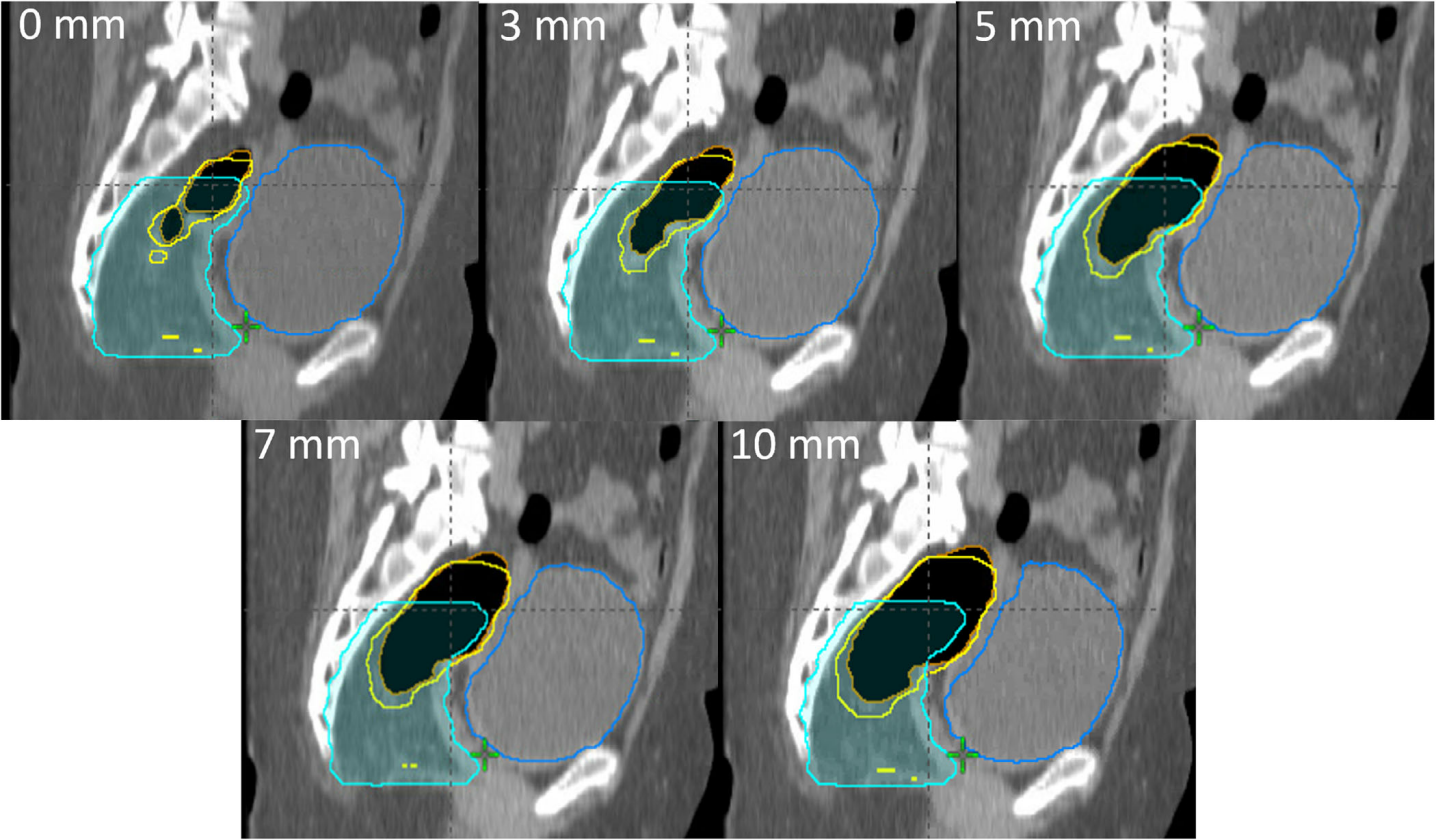
An example of a representative case demonstrating the air cavity contour (brown) and its uniform expansion from 0 to 10 mm deforming the rectum (yellow) and bladder (blue) contours relative to the high‐dose PTV contour (aqua).

Figure 2 depicts the correlation between the mean air cavity and rectum contour resulting from the expansion margins. The mean air cavity and rectum volumes for the reference plan (without expansion) were found to be 13.8 cm^3^ (range: 4.2 to 35.6 cm^3^) and 80.5 cm^3^ (range: 51.3 to 159.3 cm^3^). Upon introducing expanded air cavities of 3, 5, 7, and 10 mm, the mean air cavity volumes increased to 27.8 cm^3^ (range: 10.2 to 56.8 cm^3^), 53.3 cm^3^ (range: 22.5 to 93.5 cm^3^), 70.1 cm^3^ (range: 31.5 to 116.7 cm^3^), and 105.4 cm^3^ (range: 51.3 to 166.8 cm^3^), respectively. Similarly, with these expansions, the mean rectum volumes increased to 96.7 cm^3^ (range: 65.2 to 179.0 cm^3^), 127.4 cm^3^ (range: 84.4 to 209.8 cm^3^), 149.2 cm^3^ (range: 95.7 to 230.9 cm^3^), and 198.4 cm^3^ (range: 120.5 to 286.2 cm^3^), respectively. In each instance, the original air cavity was positioned within the high and low‐dose PTV volumes, and expansions of 3 mm and beyond were observed to intersect with or extend beyond the PTV boundary. Figure 3 illustrates a representative case in which the air cavity (brown) undergoes uniform expansion, leading to deformation of the rectum (yellow) and bladder (blue), while retaining the position of the high‐dose PTV (aqua). The original air cavity volume shown in Figure 3 is 13.5 cm^3^, which closely matches the mean volume of 13.8 cm^3^ across the twelve patients, as represented in Figure 2. It should be noted that while the simulated uniform expansion of the air cavity causes volume changes in the bladder, in a clinical scenario, the expansion would result in the displacement of the bladder without changing its volume.

### 3.2 Plan comparison

**FIGURE 4:**
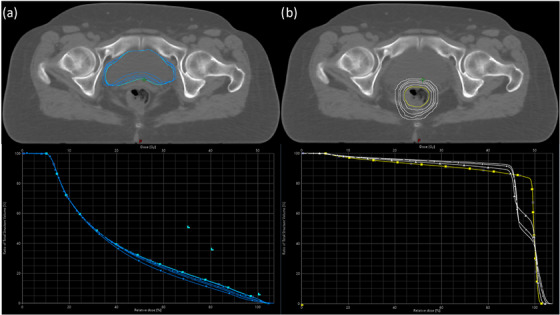
A representative case of (a) bladder contours and DVH of the reference plan (aqua) with the deformed (blue) from the expanded rectal air cavity. (b) Rectal contours and DVH of the reference plan (yellow) and the expansions (white).

Figure 4 illustrates the bladder (aqua) and rectum (yellow) contours without expansion, alongside the deformed bladder (blue) and rectum (white) contours resulting from the expanded air cavity. The figures below are the DVH curves from a representative case for these contours. Across all twelve patients, the maximum decrease in bladder dose was 5.8% ±1.4% for V35Gy, 5.5% ±1.4% for V40Gy, and 1.8% ±0.4% for V50Gy. Similarly, the rectum dose displayed a maximum reduction of 6.1% ±1.7%. These maximum reductions were observed in the plans with the largest air cavity expansion.

**FIGURE 5:**
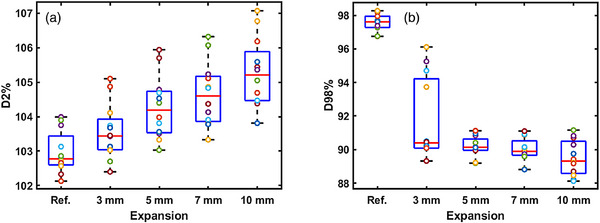
Average (a) D2% and (b) D98% in the high‐dose PTV with their respective air cavity expansion.

**FIGURE 6:**
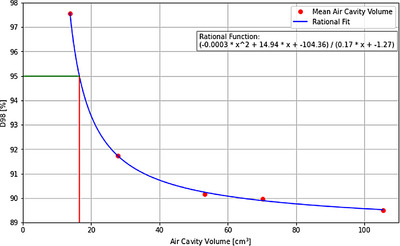
D98% rational fit for the expanded mean air cavity volume. The green horizontal line marks the 95% intercept, while the red vertical line represents the corresponding volume.

Figure 5a,b show a summary of the average D2% hotspots and D98% coverage of high‐dose PTV, respectively, alongside their corresponding air cavity expansions across all subjects. The findings indicate that with the expansion of the air cavity volume, the maximum relative change (compared to the reference plan) decreased by 10.8% ±3.5% in D98% coverage, while D2% coverage increased by 3.5% ±0.9%. A notable variability is observed particularly with the 3 mm expansion, attributable to the positioning of the air cavity. A reduction in D98% is evident when the air cavity coincides with the PTV border. On average, a 0.5% increase is observed for the D2% with each air cavity expansion. Figure 6 presents the D98% plot against the mean air cavity volume with a rational fitting function. The horizontal line indicates the dosimetry criterion of D98% ≤ 95%, while the vertical line marks the corresponding volume intercept. It was observed that when the predicted air cavity volume reached 17 cm^3^ or more, the D98% coverage fell below 95%.

**FIGURE 7:**
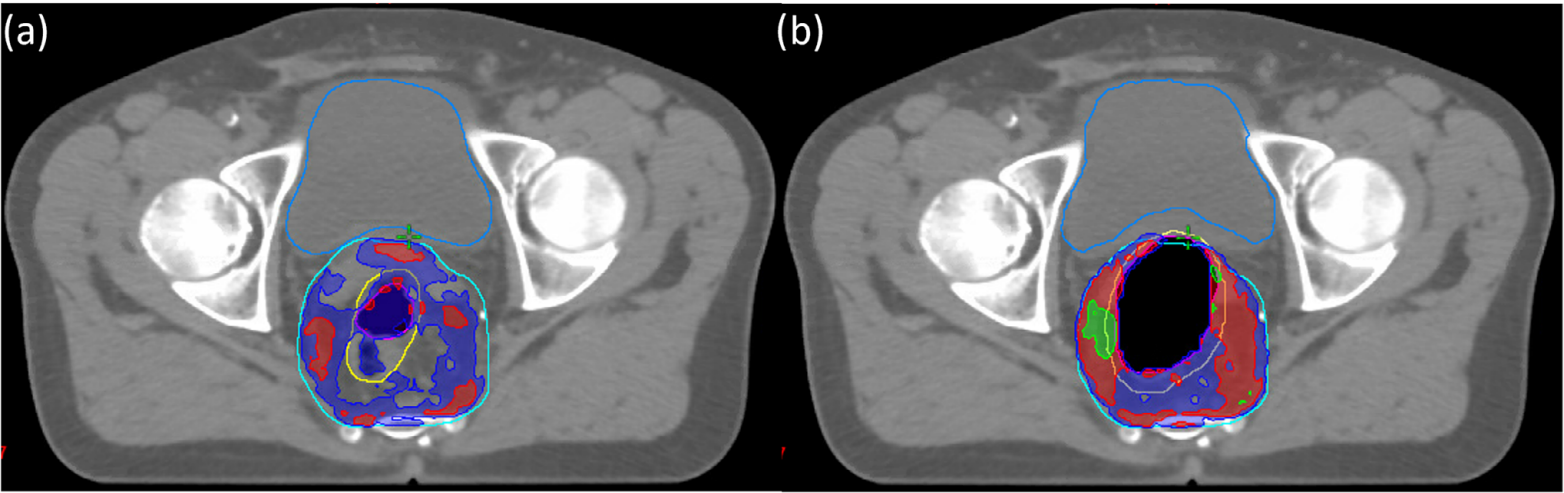
Dose color wash in the axial plane showing the isodose impact of both (a) the reference plan and (b) the 10 mm expanded air cavity within the high‐dose PTV contour (aqua). The dose color wash is represented by indicators of 100% (blue), 102% (red), and 105% (green).

Figure 7a,b depicts the dose color wash in the axial plane comparing the reference plan to the 10 mm expansion for a representative case. It was observed that as the air cavity expands, the coverage of the 102% and 105% isodoses increases, leading to larger high‐dose regions (>100%) within the plan.

## 4 DISCUSSION

In this study, we explored the influence of alterations in rectal air volume on the quality of treatment plans for patients with LARC. During radiotherapy, variations in the volume of the rectal air cavity could potentially affect the accuracy of dosimetry for the planning target volume (PTV) and nearby organs at risk (OARs), particularly the mesorectum. This scenario raises concerns regarding the potential underdosing of the mesorectum if it is displaced beyond the PTV by the presence of the air cavity. Previous studies^12^, ^14^, ^17^ noted that changes in rectal air volume affect both the PTV coverage and dose received by surrounding OAR. While pre‐treatment CBCT verification can improve the accuracy of radiotherapy delivery^23^, ^24^ by correcting patient setup errors through rigid registration between planning CT and CBCT, the effect of rectal air volume changes can alter the surrounding anatomy resulting in variation between the anatomy at planning and treatment.

It has been shown in this study that rectal air could potentially compromise PTV coverage. The original air cavities observed during CT simulation were located within both the high‐dose (50.4/50 Gy) and low‐dose (45 Gy) PTVs. Figure 3 illustrates only the high‐dose PTV, as the low‐dose PTV extends both superiorly and inferiorly. For all twelve patients, the original air cavity did not intersect the PTV contour in the anterior‐posterior or left‐right directions but may overlap between the high and low‐dose PTV contours in the superior‐inferior direction. In this region, the isodose variations are less affected by the air cavity due to the coverage provided by the low‐dose PTV, resulting in a shallower gradient compared to the anterior‐posterior and left‐right directions. The plan quality was validated using the dosimetry criterion of D98% ≥ 95%, with any coverage below 95% indicating a degraded plan. Air cavities from the original mean of 13.8 cm^3^ up to 17 cm^3^ that are contained within the high‐dose PTV did not show any plan degradation. Air cavity volumes above 17 cm^3^ can potentially degrade the high‐dose PTV coverage (D98 < 95%) while increasing the volume of the 102% and 105% isodoses. This degradation was noticed when the expanded air volume edges close to, or intersects, with the high‐dose PTV border. Daly et al^12^ noted the inclusion of a 1.5 cm margin beyond the rectal wall to ensure that the target remains within the prescription dose for 93% of the patients in their study. Our results suggest that even a 3 mm expansion of the air cavity near the PTV border adversely affects plan quality. This indicates the significance of considering both the location and size of the air cavity when evaluating CBCT during treatment.

**FIGURE 8:**
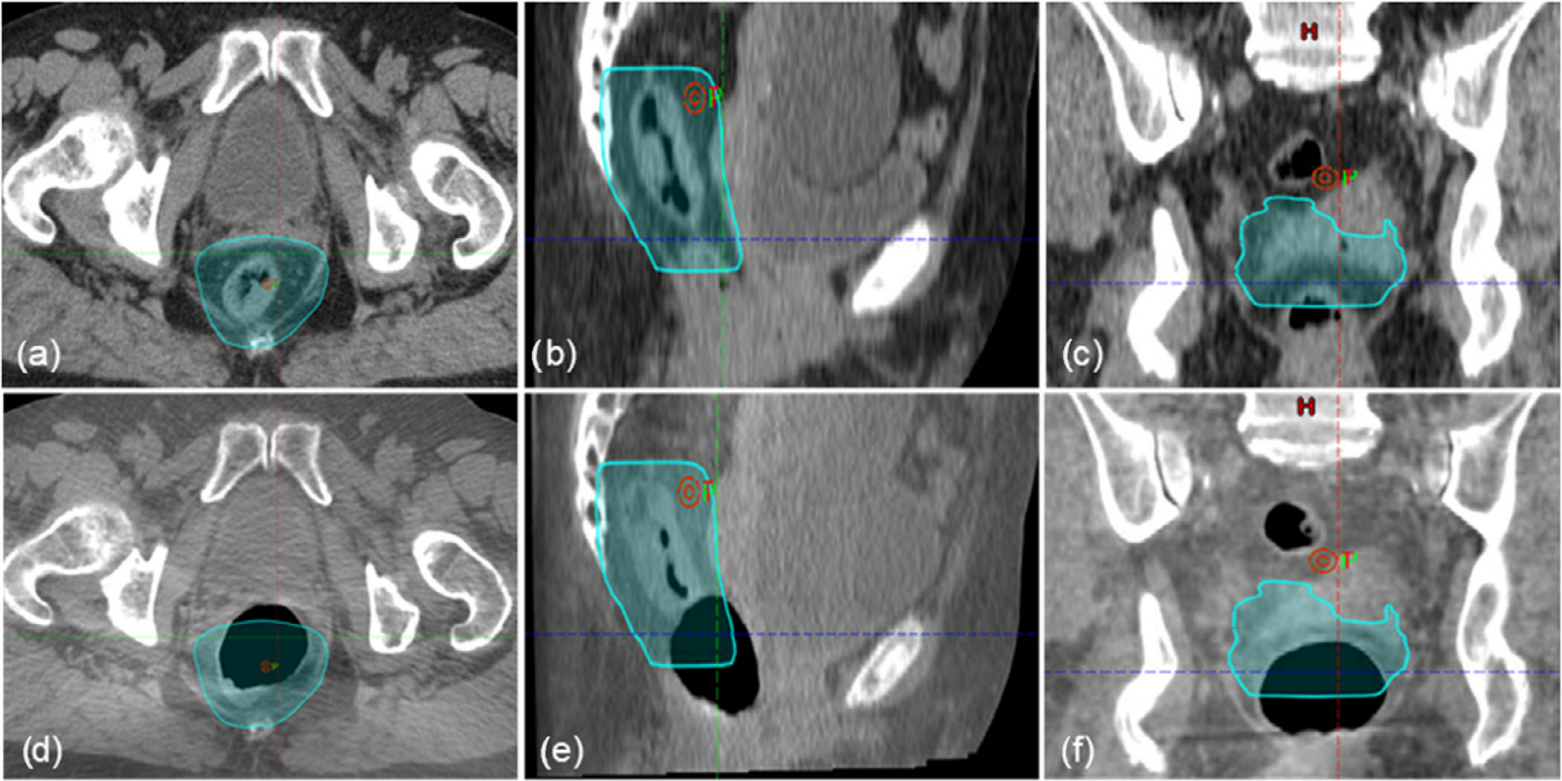
An example of a representative case planning CT in (a) axial, (b) sagittal, and (c) coronal view with the high‐dose PTV contour. The (d) axial, (e) sagittal, and (f) coronal views from a CBCT of the same patient are shown to illustrate the rectal air cavity violating the guidelines deduced from this study.

As a result of this study, clinical CBCT guidelines on acceptable rectal air volume and position during radiotherapy fractions can be deduced. Due to the inability to measure volumes during CBCT, the recommended minimum air cavity volume was converted to a spherical diameter to aid CBCT guidelines. This study has established that a maximum diameter of 3.2 cm for rectal gas in any direction (superior‐inferior, left‐right, anterior‐posterior) establishes an acceptable threshold, whereby the air cavity must not extend outside the PTV in any direction. This maximum diameter constraint can also be applied during CT simulation. By establishing CT and CBCT guidelines, patients receiving radiotherapy for LARC will have a stronger correlation between treatment plan and delivery. An example of this guideline violation is shown in Figure 8. Here a planning CT of a patient case is shown (a, b, and c) and a daily CBCT dataset showing an air cavity overlapping with the PTV (d, e, and f). By quantifying the impact an air volume may have on plan quality, we have been able to provide treatment staff with a simple method for evaluating CBCTs throughout the course of a patient's treatment, along with guidance on necessary interventions.

There are several limitations to this work. It is a retrospective study with a small number of patients and therefore findings cannot easily be generalized to all patients where the complexity of anatomical changes varies greatly. We have aimed to simulate air cavity changes and associated OAR deformations using a computational toolkit and therefore, as with all deformation studies the resulting anatomical changes may not be anatomically correct. If air is present during CT simulation and a suitable plan can be created, it is essential to evaluate changes in the air volume during treatment. In this study, we assessed the impact of expanding the initial air volume observed in the CT scans. However, we did not examine the initial air volume itself, but focused on the effects of expansions stated in this study.

## 5 CONCLUSION

The displacement of the mesorectum or perirectal space caused by the air cavity extending beyond the PTV may lead to underdosing. This study has quantified the relation between changes in rectal gas volume and radiotherapy plan quality. It has been shown that an air cavity volume exceeding 17 cm^3^ reduces PTV coverage. Based on these findings, a clinical guideline has been established, recommending a maximum threshold of 3.2 cm diameter in any direction during CBCT assessment.

## AUTHOR CONTRIBUTIONS

Eujin Chan was responsible for designing the study, collecting data, generating treatment plans, interpreting results, and writing the manuscript. Simon Goodall provided initial guidance on the study design and reviewed the analysis and interpretation of the results. Robert Finnegan handled contour deformation for all study subjects and contributed to the manuscript writing. Paul Moorfoot assisted with treatment plan generation and contour verification. Michael Jameson gave valuable feedback on the manuscript. Leon Dunn was involved in generating figures and assisting with manuscript writing. All authors reviewed the manuscript and approved the final draft for submission.

## CONFLICT OF INTEREST STATEMENT

The authors declare no conflicts of interest.
